# Body mass index as a determinant of scar formation post‐AF ablation: Insights from DECAAF II

**DOI:** 10.1111/jce.16448

**Published:** 2024-09-30

**Authors:** Ghassan Bidaoui, Eli Tsakiris, Hadi Younes, Han Feng, Ala Assaf, Nour Chouman, Mayana Bsoul, Francisco Tirado Polo, Yishi Jia, Yingshou Liu, Chanho Lim, Nadia Chamoun, Mario Mekhael, Charbel Noujaim, Amitabh C. Pandey, Swati Rao, Omar Kreidieh, Nassir F. Marrouche, Eoin Donnellan

**Affiliations:** ^1^ Tulane Research Innovation for Arrhythmia Discovery (TRIAD), Cardiac Electrophysiology Tulane University School of Medicine New Orleans Louisiana USA

**Keywords:** atrial fibrillation, atrial remodeling, catheter ablation, obesity, scar formation

## Abstract

**Introduction:**

Obesity is implicated in adverse atrial remodeling and worse outcomes in patients with atrial fibrillation. The objective of this study is to assess the effect of body mass index (BMI) on ablation‐induced scar formation on late gadolinium enhancement cardiac magnetic resonance imaging (LGE‐CMR).

**Methods:**

We conducted an analysis of DECAAF II participants who underwent LGE‐CMR scans to measure scar formation 3 months after catheter ablation. Ablation parameters and lesion delivery were not dependent on BMI. The effect of BMI on ablation success was explored.

**Results:**

Our analyses included 811 patients. Comorbidities were more prevalent in obese patients. Baseline left atrial volume was higher in obese individuals, 118, 126, 135, 140, and 143 mm^3^ for normal weight, overweight, obese grade 1, 2, and 3, respectively (*p* < .001). BMI was associated with scar formation (R = −0.135, *p* < .001), with patients with Class 3 obesity having the lowest percentage of ablation‐induced scar, 11.1%, 10.3%, 9.5%, 8.8%, 6.8% by ascending BMI group. There was an inverse correlation between BMI and the amount of fibrosis covered by ablation scar, 24%, 23%, 21%, and 18% by ascending BMI group (*p* = .001). For the fibrosis‐guided ablation group, BMI was associated with residual fibrosis (R = 0.056, *p* = .005).

**Conclusion:**

Obese patients have lower ablation scar formation, covered fibrosis, and more residual fibrosis postablation compared to nonobese patients, regardless of ablation parameters including impedance drop.

**Abbreviations**
AF: atrial fibrillation
BMI: body mass index
CA: catheter ablation
ECG: electrocardiography
LA: left atrium
LGE‐CMR: late gadolinium enhancement cardiac magnetic resonance imaging
PeAF: persistent atrial fibrillation
PV: pulmonary vein
PVI: pulmonary vein isolation

## 1 INTRODUCTION

Obesity is an important modifiable cardiovascular disease risk factor. Although body mass index (BMI) has limitations, it is the most reliable and widely used indicator of weight and obesity. Obesity is becoming a global epidemic as signified by the doubling in obesity rates in developed countries such as China, America, the United Kingdom, and India. It is projected that in around a decade, half of the population will be considered obese.^1^ Nevertheless, obesity has been associated with atrial fibrillation (AF) development through multiple disease processes. For instance, obesity increases the risk for other diseases that are known to promote AF most notably obstructive sleep apnea, diabetes, and hypertension. Furthermore, obesity is correlated with hemodynamic and metabolic changes such as an increase in inflammatory and profibrotic markers that are known to cause structural and electrophysiological changes in the atrium, effectively leading to atrial myopathy.^1^, ^2^, ^3^ Finally, increased pericardial volume and epicardial fat has been shown to be associated with AF progression and worse outcomes with treatment.^4^, ^5^

AF is a complex arrhythmia that usually progresses from paroxysmal to a permanent form. The development and progression of AF is dependent on increased structural and electrical remodeling in the left atrium (LA).^1^ It is hypothesized that the main trigger for the AF episodes is the muscular sleeve within the pulmonary vein (PV) ostia. Extra‐PV triggers and substrate for AF perpetuation lead to more sustained and uncontrolled disease in patients with advanced disease. Structural changes in the myocardium consist of atrial fibrosis and atrial enlargement.^6^ These changes can be assessed by CMR with studies showing the negative prognostic impact on treatment success in these patients.^7^

Catheter ablation (CA) has emerged as an effective treatment of AF as it targets the main pathophysiological mechanisms associated with AF and induces either the slowing or even regression of the myopathy.^8^ Pulmonary vein isolation (PVI) has been shown to be the most important aspect determining CA success. However, the success rate of PVI drops significantly in persistent atrial fibrillation (PeAF), possibly secondary to increased extra‐PV triggers and adverse atrial remodeling. Late gadolinium enhancement cardiac magnetic resonance imaging (LGE‐CMR) has been shown to reliably measure atrial fibrosis, a major nidus for re‐entry currents.^9^ The extent of atrial fibrosis, as detected by LGE‐CMR, has been shown to be associated with CA outcomes, regardless of the ablation strategy.^7^ Targeting this extra‐PV substrate on CMR has not been shown to increase ablation success in PeAF patients.^10^ LGE‐CMR has also been demonstrated to be useful for the assessment of scar formation by CA postablation, a major determinant of treatment success.^11^ Nevertheless, the factors associated with scar formation are not well‐elucidated, especially in PeAF patients. In this subanalysis, we are going to explore the effect of BMI on ablation‐induced scar formation in PeAF patients.

## 2 METHODS

### 2.1 Study design

This study is a subanalysis of the DECAAF II^10^ trial evaluating the effect of BMI on atrial myopathy, remodeling, and ablation outcomes in PeAF patients. A full discussion of the DECAAF II methods has been described previously in the literature. The DECAAF II trial was a prospective, randomized controlled, investigator‐initiated, industry‐sponsored, multicenter, single‐blinded trial that randomized PeAf patients to receive standard PVI or PVI plus fibrosis‐guided ablation. Patients were recruited from July 2016 through January 2020. Of the 843 randomized patients, 422 patients were in the PVI arm with the rest included in the intervention arm (421 patients). Patients followed up for 12–18 months including the blanking period using daily single‐lead smartphone electrocardiography (ECG) recordings (ECG Check Device, Cardiac Designs Inc.) to assess for recurrence. Patients were also required to send a strip to the ECG core laboratory if they experienced any concerning symptoms in the follow‐up period. Ambulatory monitoring and 12‐lead ECG data performed as part of clinical care were also included.

### 2.2 Patient population

**Figure 1:**
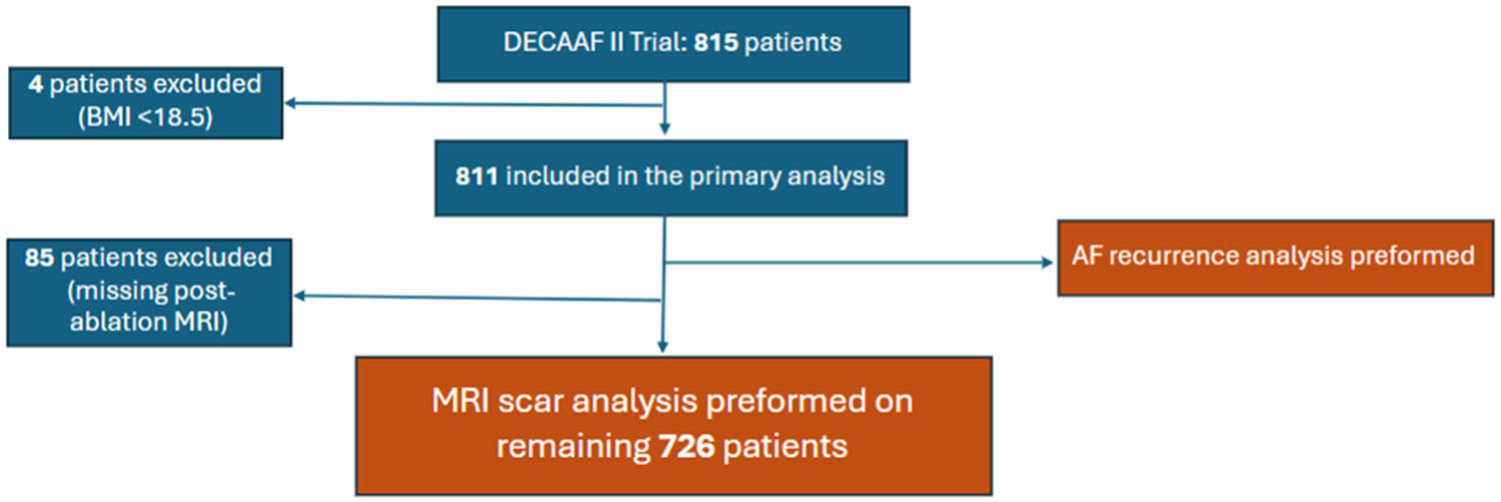
Patient selection and analysis cohorts. This schematic demonstrates the sequential exclusion criteria and the cohorts with which the various analyses were performed. Patients with BMI < 18.5 were excluded given their small sample size. BMI, body mass index.

To enroll, patients had to be at least 18 years old and have documented PeAF without prior ablation. PeAF was defined as lasting 7 days or more on a rhythm strip or chart review. Exclusion criteria included patients with prior ablation or valvular cardiac surgery, contraindication for CMR or the use of beta blockers, inability to be positioned appropriately in the CMR scanner, pregnant women, terminally ill, or without smartphone capability. In this subanalysis, patients with BMI readings above 18.5 were included in the study for assessment of comorbidities and ablation outcomes such as recurrence. Patients with a BMI < 18.5 were excluded given their small sample size compared to other groups (*n* = 4). Normal BMI (18.5–24.9) is used as the reference group in this analysis. For ablation scar formation analysis and LA remodeling, only patients with BMI readings and two CMRs available (pre‐ and postablation) were included (Figure 1).

### 2.3 Imaging protocol

All participants underwent LGE‐CMR within 30 days before their ablation procedure and again between 90 and 180 days after. In cases where a patient's heart rate exceeded 90 beats per minute at the time of the MRI scan, beta blockers were administered beforehand. Approximately 15 min following the administration of the contrast agent, images were obtained using a high‐resolution, three‐dimensional, inversion‐recovery‐prepared, ECG‐gated, respiratory‐navigated, gradient‐echo pulse sequence for LGE‐CMR. The timing of image capture was carefully chosen to occur before atrial systole, during a period known to have minimal LA wall movement, ensuring clearer images. The Merisight delayed CMR protocol by MARREK Inc was used for imaging processing. The images were sent to MARREK Inc for processing, evaluation, and quantification in relation to the preablation baseline LA fibrosis and the postablation LA scar formation. The segmentation of the LA wall was done manually, and to identify atrial fibrosis, an intensity threshold was applied, ranging from two to three standard deviations above the average intensity of the normal tissues, as determined by expert review of each image. The processing and segmentation processes were uniform for all the imaging done in the study and performed by MARREK. Baseline fibrosis measurement involved calculating the fibrotic surface area in the LA wall before ablation and dividing it by the LA wall's surface area. The postablation scar area was quantified by the percentage of the LA surface showing enhancement at a heightened LGE threshold, concealing baseline fibrosis, thus representing the iatrogenic scar from CA. The CMR images from before and after ablation were initially aligned using affine registration for detailed examination, followed by deformable registration. A 3D map of LA fibrosis was then constructed with the Corview volume rendering tool. The areas on the CMR indicating baseline fibrosis that overlapped with the postablation scar were identified as covered fibrosis, whereas those not overlapped by scar were termed noncovered fibrosis. Residual fibrosis and noncovered fibrosis refer to the same areas on the CMR, with the residual fibrosis having the whole LA surface as the denominator and the noncovered fibrosis having the baseline fibrosis as the denominator.

### 2.4 Ablation technique

Patients randomized to the PVI arm underwent the procedure as described by the Heart Rhythm Society Consensus Statement. All PVs were electrically isolated and if normal sinus rhythm could not be restored even after cardioversion, the operator can pursue further ablation including the addition of line to eliminate recurrent arrhythmias as needed. As for the fibrosis‐guided arm, LGE‐CMR images were merged with the 3D mapping system to be used during the procedure. All patients first underwent PVI, then PV entrance block was confirmed. Then, operators either encircled or covered all fibrotic areas observed on CMR with ablation lesions. CA was performed through radiofrequency or cryoablation.

### 2.5 Statistical analysis

All continuous variables are presented as mean ± standard deviation and compared using Student's *t* tests and Mann–Whitney tests, according to the results of normality assumption check through Shapiro–Wilk test. Categorical variables are presented as percentages or frequencies and compared using Chi‐Square tests or Fisher tests. Univariable and multivariable linear models were developed to identify predictors of the formation of ablation scar, covered fibrosis, and noncovered fibrosis and the results are reported as regression coefficients (beta). Variables in the multivariable models were selected based on clinical utility and confounding potential based on the literature. If two variables were found to be strongly collinear, only the variable with a stronger coefficient is included in the model. Furthermore, the patients were divided into normal weight, overweight, and three increasingly obese classes based on BMI ranges presented by the centers for disease control and prevention (CDC). All statistical analyses were performed using R 4.2.0 (The R Foundation)^12^ or SPSS 29 with a two‐sided significance level of 0.05 by default.

## 3 RESULTS

### 3.1 Baseline characteristics

Our study evaluated 811 individuals from DECAAF II trial who had a BMI greater than or equal to 18.5 kg/m^2^. The mean age of the participants was 63 years old and was majority male (79.3%). The patients in all groups were distributed according to the treatment received. The prevalence of comorbid conditions such as hypertension, hyperlipidemia, diabetes mellitus, and history of stroke was higher in obese groups, although the prevalence of other comorbidities such as congestive heart failure and coronary artery disease did not differ significantly (Table 1).

**Table 1:** Baseline characteristics, demographics by obesity class by BMI.

Variable	Overall	Normal weight (18.5–24.9)	Overweight (25–29.9)	Obese Class 1 (30–34.9)	Obese Class 2 (35–39.9)	Obese Class 3 (≥40)	*p* Value
Number of patients	811	87	268	267	123	66	
Age (years)	811	63 (10)	63 (9)	62 (9)	62 (8)	60 (9)	
Sex	811						.03
Female	169	25 (29%)	54 (20%)	46 (17%)	23 (19%)	21 (32%)	
Male	642	62 (71%)	214 (80%)	221 (83%)	100 (81%)	45 (68%)	
BMI	811	23.2 (1.5)	27.7 (1.4)	32.3 (1.4)	36.8 (1.4)	45.3 (6.8)	
PVI + fibrosis‐guided ablation	811	45 (52%)	138 (51%)	126 (47%)	58 (47%)	37 (56%)	.60
Congestive heart failure	811	17 (20%)	44 (16%)	50 (19%)	29 (24%)	13 (20%)	.60
Hypertension	811	32 (37%)	131 (49%)	164 (61%)	99 (80%)	53 (80%)	<.001
Diabetes mellitus	811	4 (4.6%)	19 (7.1%)	27 (10%)	21 (17%)	11 (17%)	.004
History of stroke	811	12 (14%)	21 (7.8%)	17 (6.4%)	16 (13%)	2 (3.0%)	.03
Smoking	811	32 (37%)	95 (35%)	107 (40%)	45 (37%)	23 (35%)	.80
Coronary artery disease	811	8 (9.2%)	35 (13%)	25 (9.4%)	22 (18%)	10 (15%)	.13
Mitral valve disease	811	8 (9.2%)	18 (6.7%)	16 (6.0%)	4 (3.3%)	2 (3.0%)	.40
Hyperlipidemia	811	16 (18%)	81 (30%)	97 (36%)	53 (43%)	26 (39%)	.002
Antiarrhythmic Medicationsm	811	9 (10%)	39 (15%)	39 (15%)	12 (9.8%)	11 (17%)	.50
Beta‐blockers	811	60 (69%)	195 (73%)	204 (76%)	94 (76%)	52 (79%)	.50
Calcium channel blockers	811	9 (10%)	34 (13%)	69 (26%)	44 (36%)	19 (29%)	<.001
ACE inhibitors	811	17 (20%)	78 (29%)	77 (29%)	36 (29%)	25 (38%)	.20
ARB inhibitors	811	14 (16%)	55 (21%)	70 (26%)	45 (37%)	23 (35%)	<.001
Aldosterone inhibitors	811	4 (4.6%)	17 (6.3%)	22 (8.2%)	13 (11%)	8 (12%)	.30
Statins	811	22 (25%)	82 (31%)	101 (38%)	46 (37%)	22 (33%)	.20
Anticoagulation	811	83 (95%)	257 (96%)	257 (96%)	118 (96%)	64 (97%)	.90
History of cardioversion	811	17 (20%)	50 (19%)	45 (17%)	28 (23%)	14 (21%)	.70
Failed antiarrhythmic treatment	811	46 (53%)	152 (57%)	157 (59%)	72 (59%)	39 (59%)	.90

Abbreviations: ACE, angiotensin‐converting enzyme; ARB, angiotensin receptor blocker; BMI, body mass index; PVI, pulmonary vein isolation.

### 3.2 CMR indices

**Figure 2:**
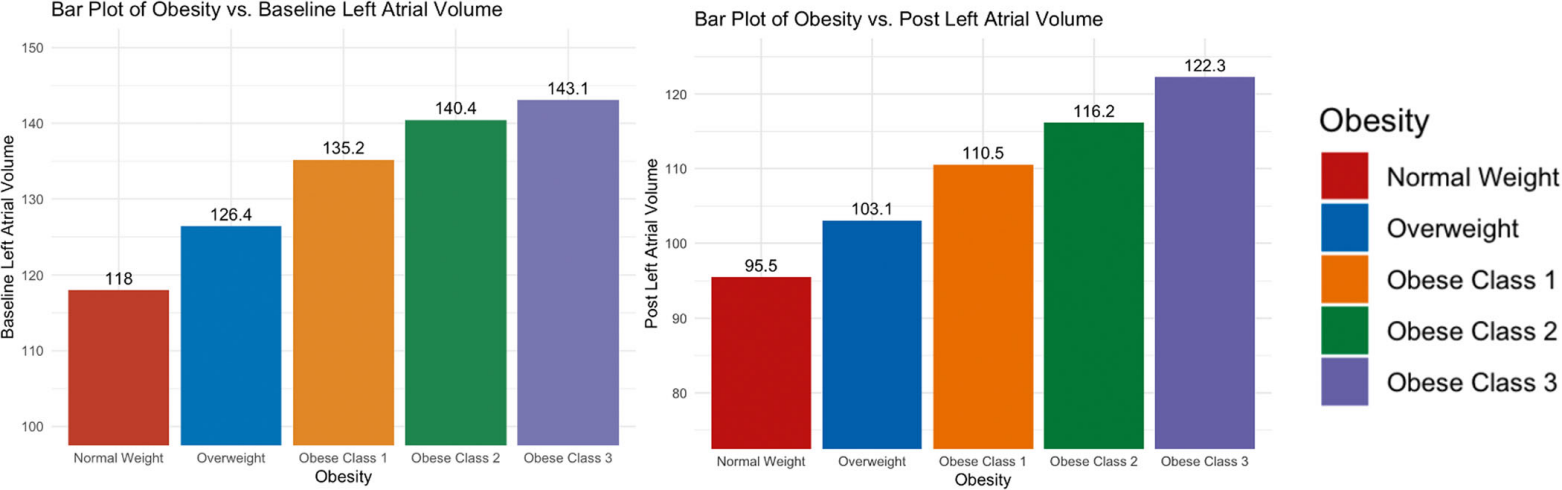
Higher left atrial volume with obesity. This figure demonstrates the observed gradual increase in mean baseline left atrial volume (A) and postablation left atrial volume (B) among groups stratified based on obesity category.

Of the 811 patients selected for analysis, only 726 underwent postablation LGE‐CMR in the trial. Hence, the evaluation for atrial remodeling and scar formation was limited to these patients given the availability of data. As shown in Table 2, BMI was not associated with baseline fibrosis levels. However, the LA volume was directly correlated with BMI and significantly higher pre‐ and postablation in obese patients (Figure 2). LA volume change was not significantly different among the different groups.

**Table 2:** Baseline characteristics, LGE‐CMR indices, and ablation parameters based on obesity class by BMI.

Variable mean (SD)	Normal weight (18.5–24.9)	Overweight (25–29.9)	Obese Class 1(30–34.9)	Obese Class 2(35–39.9)	Obese Class 3 ( ≥ 40)	*p* Value
Days from CMR to ablation	16 (21)	19 (54)	18 (37)	17 (26)	12(11)	.8
Days from ablation to postablation CMR	234 (240)	186 (163)	172 (111)	175 (127)	190 (172)	.2
Days from AF diagnosis to ablation	1123 (1641)	1136 (1487)	811 (1123)	872 (1184)	760 (1198)	.05
Baseline fibrosis (%)	19 (7)	19 (8)	19 (7)	20 (8)	19 (7)	.5
Baseline LA volume (mm^3^)	118 (43)	126 (38)	135 (41)	140 (40)	143 (42)	<.001
Ablation scar (%)	11.1 (5.2)	10.3 (5.1)	9.5 (5.0)	8.8 (4.9)	6.8 (3.4)	<.001
Noncovered fibrosis (%)	76 (11)	77 (12)	79 (11)	79 (10)	82 (8)	.001
Covered fibrosis (%)	24 (11)	23 (12)	21 (11)	21 (10)	18 (8)	.001
Residual fibrosis (%)	14 (6)	14 (6)	15 (6)	16 (7)	16 (6)	.2
Postablation LA volume (mm^3^)	96 (33)	103 (32)	111 (36)	116 (36)	122 (40)	<.001
Change in LA volume (mm^3^)	21 (24)	23 (25)	25 (27)	23 (29)	19 (31)	.4
Sheath time	156 (62)	162 (74)	168 (79)	153 (63)	169 (69)	.2
Ablation points	126 (86)	124 (78)	135 (89)	120 (77)	147 (109)	.6
Mean maximum power used	32 (6)	41 (75)	38 (64)	47 (118)	34 (8)	.7
Mean contact force used	16.7 (8.4)	16.1 (5.2)	15.5 (4.7)	14.2 (4.3)	14.4 (4.1)	.2
Impedance drop	8.21 (2.97)	8.51 (2.98)	8.10 (2.70)	7.70 (2.56)	7.17 (2.33)	.031

Abbreviations: AF, atrial fibrillation; BMI, body mass index; LGE‐CMR, late gadolinium enhancement cardiovascular magnetic resonance; LA, left atrial/atrium.

**Figure 3:**
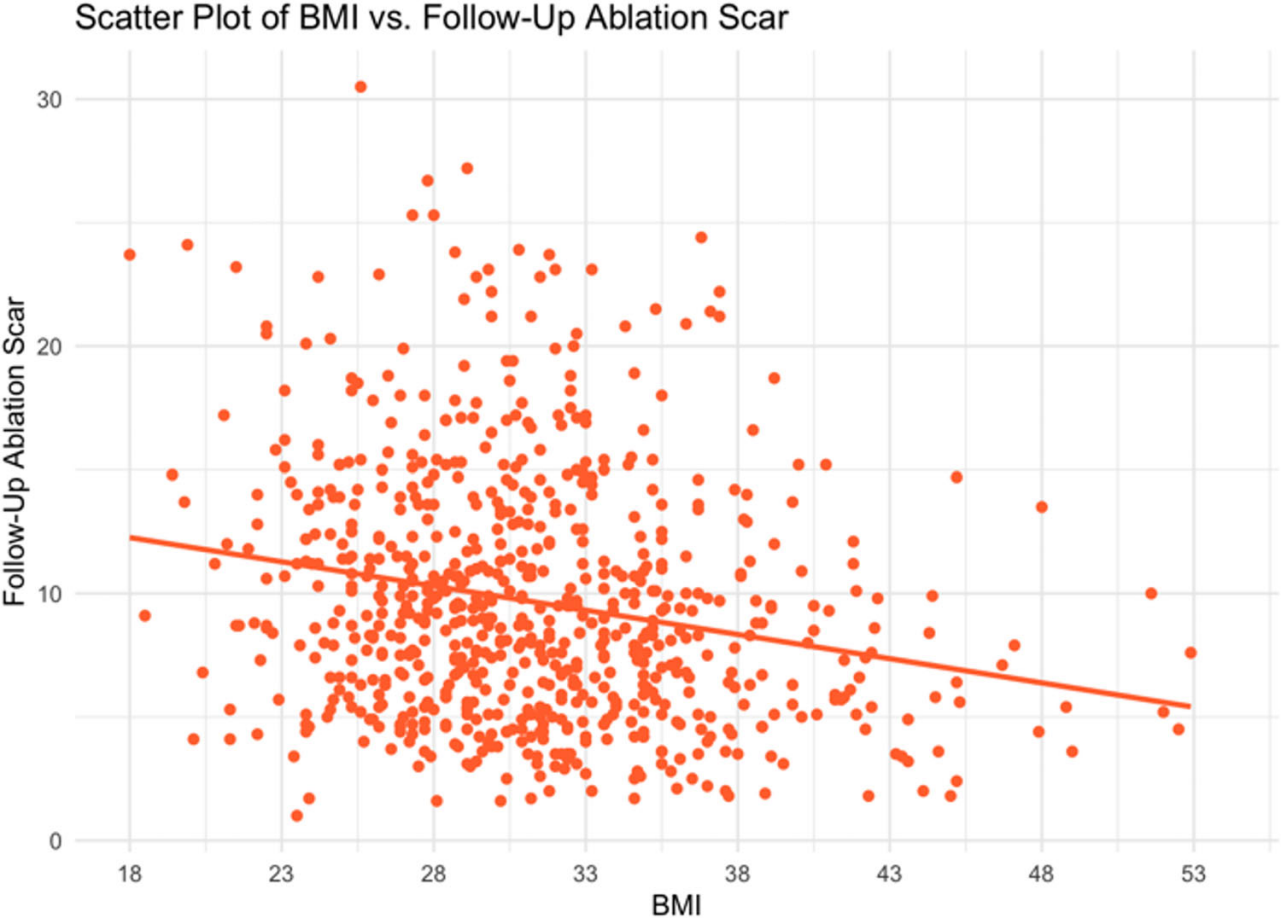
BMI Negatively correlated with follow‐up ablation scar. This scatter plot demonstrates the observed negative correlation between the size of the follow‐up ablation scar and a patient's BMI. BMI, body mass index.

**Figure 4:**
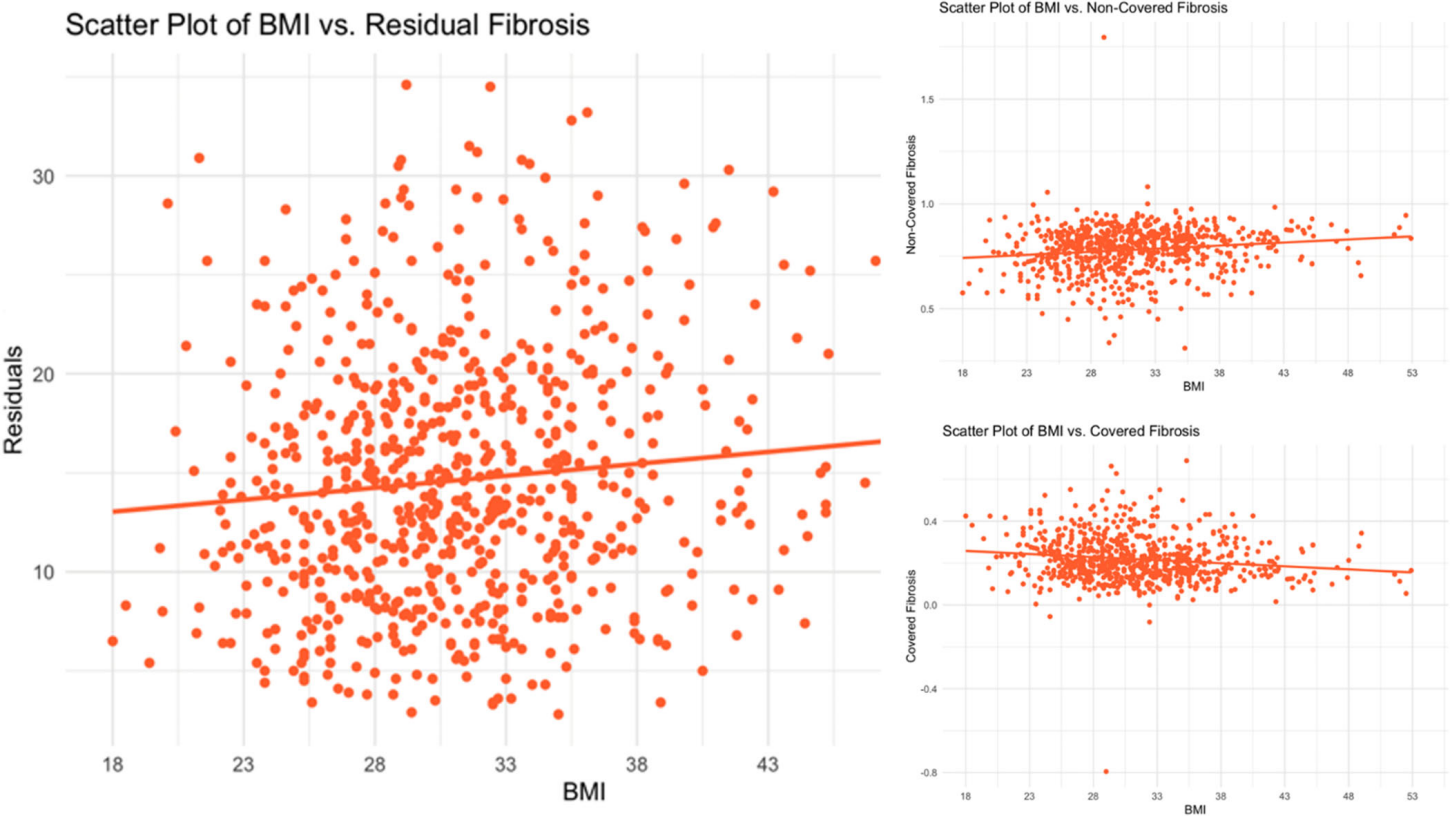
BMI positively correlated with residual fibrosis. Left: BMI is positively correlated with the amount of residual fibrosis. By extension, the amount of noncovered fibrosis (top, right) is higher, and the amount of covered fibrosis (bottom, right) is lower, in patients with higher BMI. BMI, body mass index.

With respect to ablation‐induced scar formation, a significant negative correlation was found between BMI as a continuous variable and scar formed (R = −0.135, *p* < .001), with ablation scar decreasing stepwise from 11.1% in the normal weight group to 6.8% in the Class 3 Obese group (Table 2, Figure 3). As shown in Table 2, patients across all groups were ablated equally with similar contact force, power used, number of ablation tags, and ablation time (*p *> .05). Importantly, impedance drop was the only factor that differed and is known to reflect tissue characteristics (*p *> .05). Figure 3 demonstrates postablation scar formation and fibrosis coverage with respect to BMI as a continuous variable in the entire study cohort. We found that BMI affects the follow‐up ablation scar, and by extension, is negatively correlated with scar‐covered fibrosis (R = −0.135, *p* < .001), and positively correlated with residual fibrosis (R = 0.052, *p* = .003) (Figure 4). This correlation was examined in a multivariable fashion in both treatment arms and the correlation remained significant in the whole population (R = −0.135, *p* = .002), PVI‐only arm (R = −0.124, *p* = .017), and the fibrosis‐guided arm (R = −0.148, *p* = .04). The multivariable analyses are shown in Table 3 and the Supporting Information.

**Table 3:** Postablation scar formation and fibrosis coverage, multivariate analysis.

Follow‐up ablation scar with multiple covariates			
	Coefficient	Standard error	*p* Value
(Intercept)	7.289	2.692	.007
BMI	−0.135	0.044	.002
Age	0.058	0.030	.057
Male gender	0.864	0.666	.196
LA volume	−0.011	0.007	.108
Baseline fibrosis	0.027	0.037	.460
C‐reactive protein	−0.091	0.041	.027
Diabetes mellitus	0.133	0.942	.888
Congestive heart failure	1.712	0.656	.010
Hypertension	0.456	0.556	.412
Coronary artery disease	0.421	0.994	.672
Peripheral vascular disease	−1.160	1.107	.296
History of stroke	0.915	1.018	.369
Use of aldosterone inhibitors	−1.067	0.892	.233
History of antiarrhythmics	0.505	0.503	.316
PVI + fibrosis‐guided ablation	2.420	0.499	<.001
Impedance drop	0.226	0.095	.018

Residual fibrosis with multiple covariates			
	Coefficient	Standard error	*p* Value
(Intercept)	−0.912	1.147	.427
BMI	0.052	0.019	.005
Age	−0.006	0.013	.645
Male gender	0.061	0.284	.831
LA volume	−0.002	0.003	.611
Baseline fibrosis	0.825	0.016	<.001
C‐reactive protein	0.023	0.018	.193
Diabetes mellitus	0.238	0.401	.554
Congestive heart failure	−0.190	0.280	.498
Hypertension	−0.162	0.237	.494
Coronary artery disease	−0.326	0.423	.442
Peripheral vascular disease	0.644	0.472	.173
History of stroke	−0.371	0.434	.392
Aldosterone inhibitor use	0.102	0.380	.788
History of antiarrhythmic medication	−0.156	0.214	.466
PVI + fibrosis‐guided ablation	−0.823	0.212	<.001
Impedance drop	−0.053	0.041	.195

Covered fibrosis with multiple covariates			
	Coefficient	Standard error	*p* Value
(Intercept)	0.271	0.069	<.001
BMI	−0.003	0.001	.013
Age	3.67E‐04	0.001	.635
Male gender	−0.007	0.017	.662
LA volume	1.53E‐04	0.000	.391
Baseline fibrosis	−0.003	0.001	.001
C‐reactive protein	−0.001	0.001	.295
Diabetes mellitus	−0.018	0.024	.460
Congestive heart failure	0.016	0.017	.352
Hypertension	0.008	0.014	.575
Coronary artery disease	0.016	0.025	.518
Peripheral vascular disease	−0.031	0.028	.272
History of stroke	0.013	0.026	.622
Aldosterone inhibitor use	1.16E‐04	0.023	.996
Antiarrhytmic medications	0.005	0.013	.704
PVI + fibrosis‐guided ablation	0.048	0.013	<.001
Impedance drop	0.004	0.002	.094

Abbreviations: BMI, body mass index; LA, left atrial/atrium; PVI, pulmonary vein isolation.

### 3.3 Recurrence

**Figure 5:**
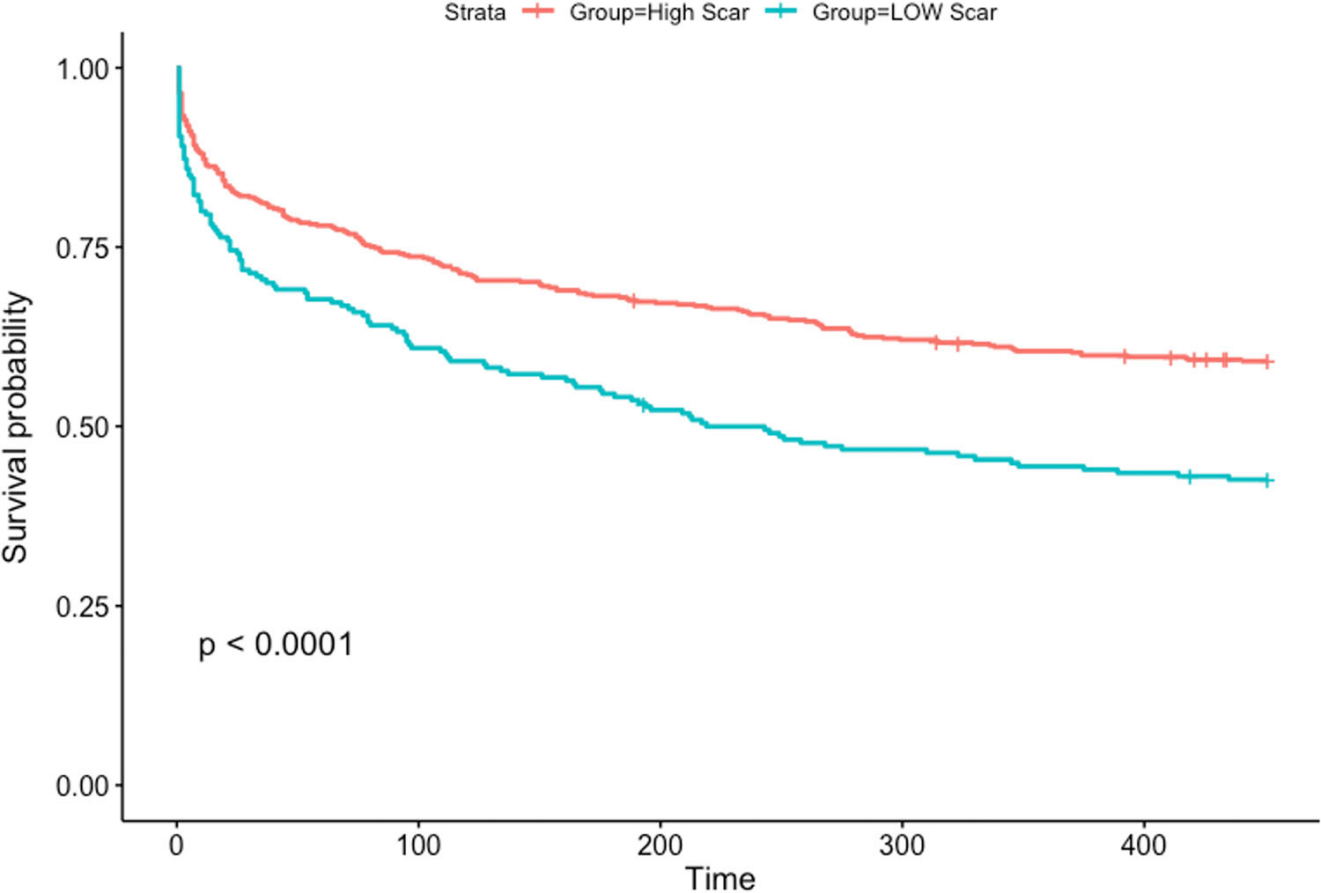
Kaplan–Meier curve of survival from AF recurrence for patients with high versus low scar. The cutoff of 6.5% scar formation is used above to delineate two groups, high and low scar. The group with high scar formation demonstrates a statistically significant longer time to recurrence following ablation. AF, atrial fibrillation.

**Figure 6:**
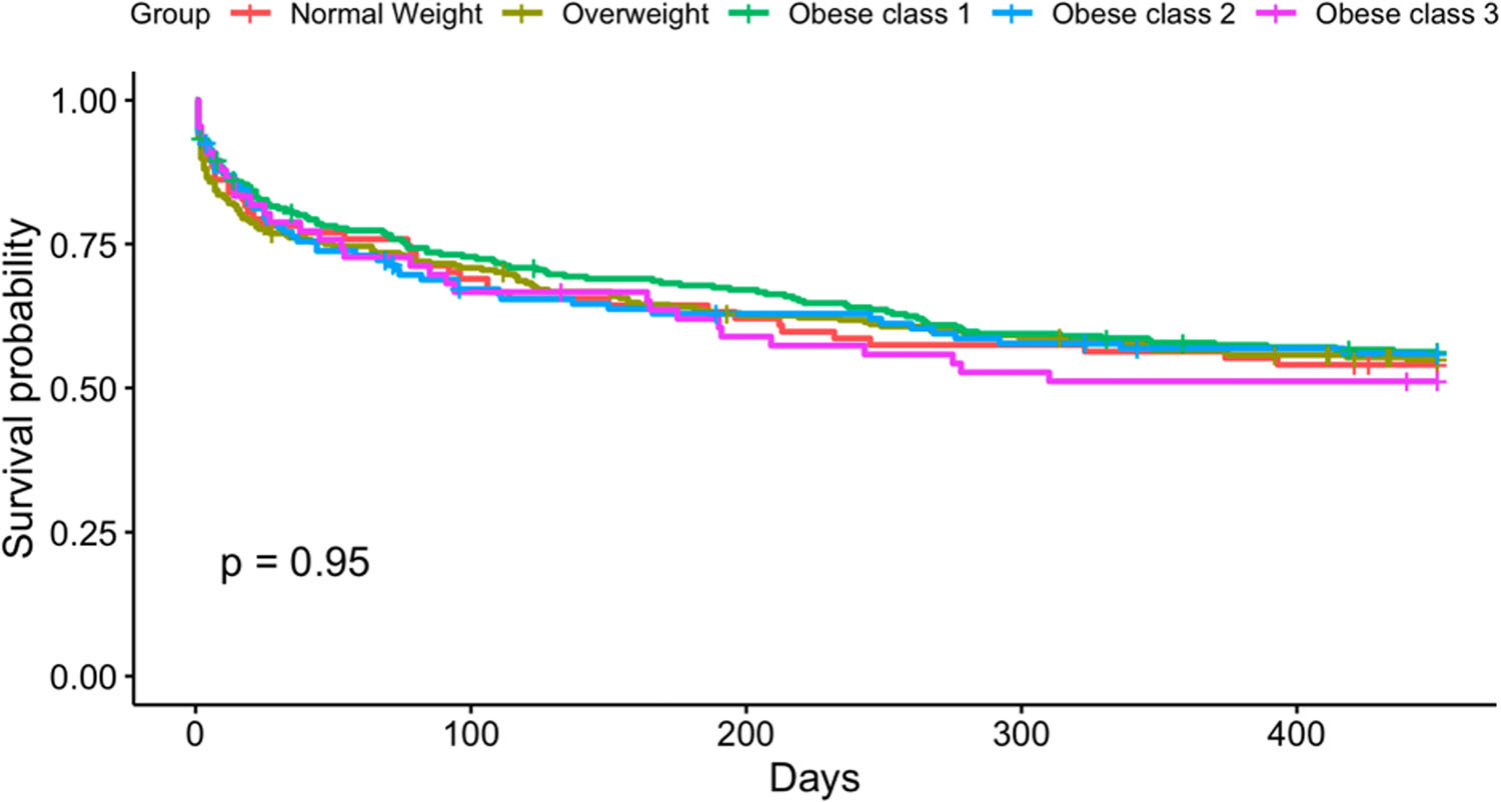
Kaplan–Meier curve of AF recurrence by BMI class. No significant difference in time‐to‐recurrence among the obesity subgroups. AF, atrial fibrillation; BMI, body mass index.

To assess the effect of scar formation after CA and fibrosis coverage on ablation success, an unadjusted analysis was performed. This demonstrated that larger postablation scar is associated with a reduced risk of AF recurrence (R = 0.97, *p* = .016). Our analysis also showed that scar formation above 6.5% in the PVI group and 6.23% in the fibrosis‐guided group best differentiated who is going to recur after the procedure (*p* < .0001, Figure 5). Patients with a high‐level scar formation based on these cut‐offs had a lower BMI compared to patients with unfavorable scar formation. (30.8 ± 5.7 vs. 33.4 ± 7.2, *p* < .001). Regarding the direct relationship between BMI and AF recurrence, no significant correlation was found (confidence interval: 0.982–1.015; *p* = .849, Figure 6).

## 4 DISCUSSION

This subanalysis of the DECAAF II trial patients led to several important findings regarding the association between BMI and ablation‐induced scar formation in our PeAF cohort. First, we confirmed the importance of ablation scar formation to ablation success in PeAF patients. Second, BMI is a significant negative predictor of ablation‐induced scar formation both in the PVI arm and the fibrosis‐guided ablation arm regardless of ablation parameters such as contact force, power, number of ablation points, and impedance drop. Third, BMI affected substrate modification, as demonstrated by covered fibrosis and residual fibrosis, in the fibrosis‐guided arm. Fourth, BMI was associated with increased LA volume at baseline and 3 months postablation. Finally, even though a higher BMI impacts scar tissue formation and obese patients had more comorbidities, obesity was not associated with a higher rate of the procedure's failure, as judged by recurrence of AF.

The association between formed scar and success was assessed, confirming the negative correlation between scar formed and recurrence.^13^ Although ablation of atrial tissue is done to form stable and durable scars, this is not achieved consistently on all ablation points.^14^, ^15^ The formation of durable scar depends on multiple factors including the extent of atrial myopathy and ablation parameters. In this cohort, a scar percentage of around 6.31 was found to be associated with increased success rate of ablation. The cut‐off was found to be different between patients who underwent PVI only and patients who underwent CMR fibrosis‐guided ablation, which is expected given the smaller surface area on which ablation was planned in the PVI group and the higher number of ablation points expected in the fibrosis‐guided ablation group.

BMI was consistently associated with worse scar formation in patients who underwent PVI or PVI plus extrapulmonary substrate modification. The relationship between BMI and AF development and maintenance is well‐known, although the mechanistic explanation of this relationship is not straightforward. That said, the effect of BMI on CA outcomes in patients with advanced disease is even more complex. In our analysis, a 1% increase in BMI was associated with a 0.135% decrease in ablation‐induced scar formation. This can serve as a demonstration and explanation of the effect of BMI on atrial myopathy, response, and success of ablation. For instance, one possible explanation is the higher rate of comorbidities in these patients, leading to blunted remodeling with CA. However, even after controlling for relative confounder and comorbidities including diabetes, coronary artery disease, and heart failure, BMI was still associated with worse scar formation, hinting toward possible underlying biophysical factors affecting durable scar formation in the obese cohort.

**Figure 7:**
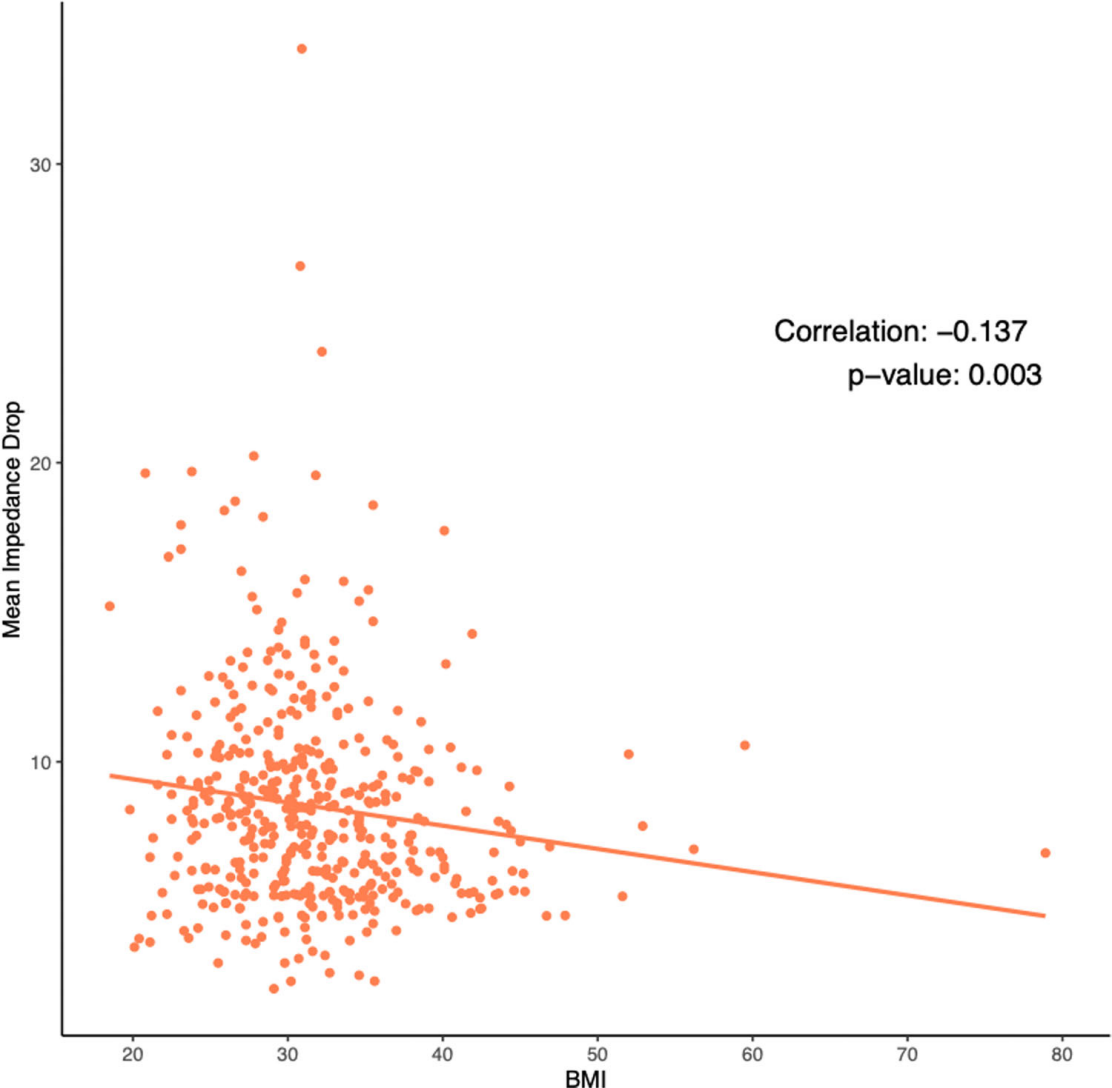
Impedance drop correlation with BMI. Impedance drop measured during ablation is found to be negatively correlated with BMI. BMI, body mass index.

To assess the possible implications of worse scar formation on substrate modification with increased BMI, we assessed the coverage of the atrial substrate with scar. Fibrosis modification and coverage was found to also be affected by BMI. BMI was not part of the criteria listed in the ablation of these areas. Interestingly, contact force, maximum ablation power, number of ablation points, and sheath time was not different among the groups. Yet, given the blunted scar formation associated with BMI in our cohort, fibrosis coverage was also affected. The ALICIA and DECAAF II trials did not show a positive impact of attacking substrate as assessed by CMR.^10^, ^16^ The association of BMI with the amount of scar formed, not reported previously, illustrates the complexities associated with covering the substrate effectively and inducing durable scar. For example, some studies showed that acute edema and scar induced by ablation decreases by 64% on the long‐term.^17^ Moreover, the extent of atrial myopathy and its effect of scar durability has not been well‐elucidated. Nevertheless, one of the possible factors that could have affected ablation scar in obese patients is the difference in impedance characteristics associated with obesity. Higher baseline impedance and less impedance drop is expected with increasing BMI which might guide ablation in these patients.^18^, ^19^ Surprisingly, although impedance drop was associated with BMI in our cohort (Figure 7), its effect did not neutralize the deleterious effect BMI has on scar formations.^20^ Furthermore, baseline impedance was not found to be altered based on BMI (Central Illustration 1).

**Central Illustration 1:**
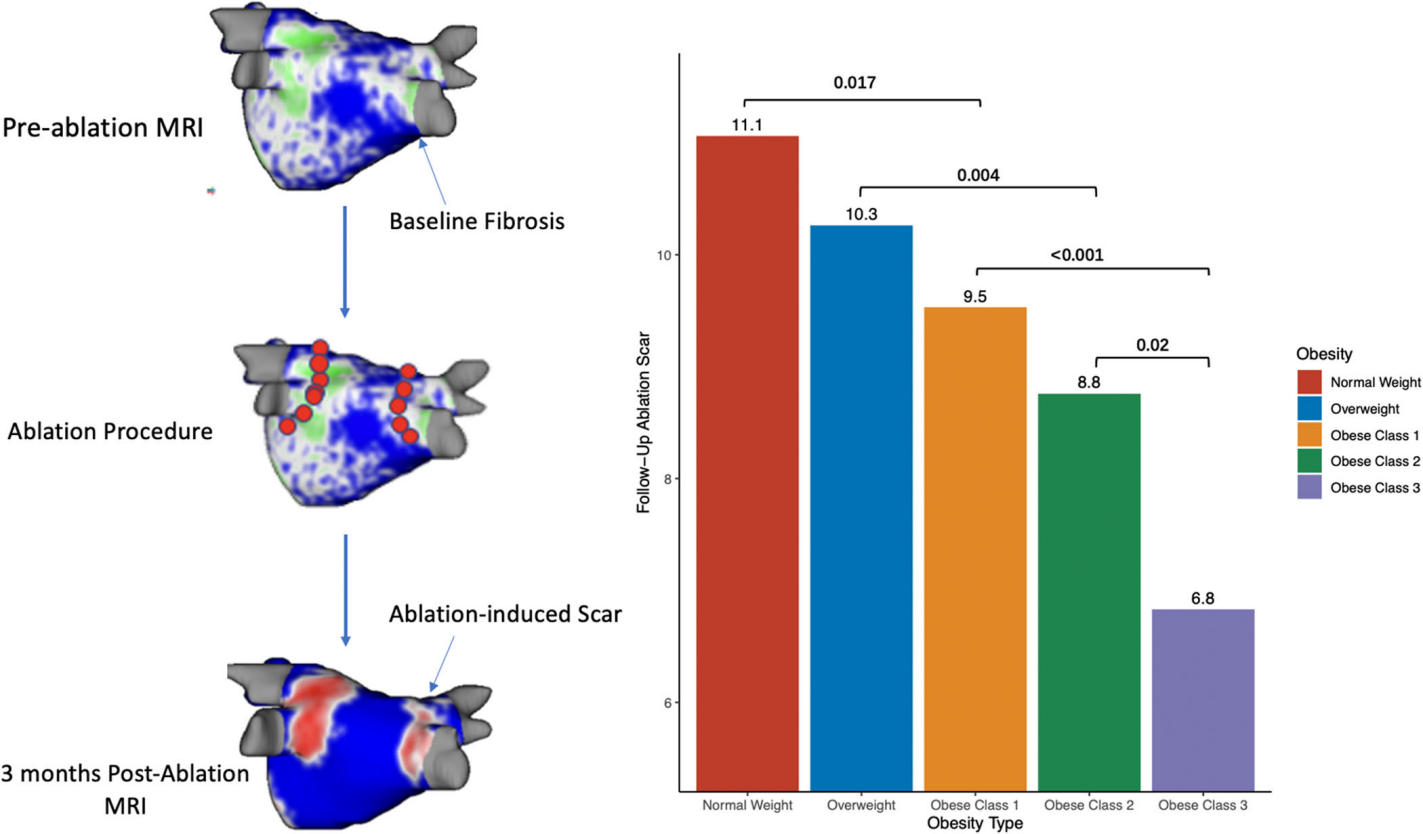
Obesity is associated with reduced catheter ablation‐induced scar formation on late‐gadolinium enhancement MRI.

Another atrial myopathy marker on CMR is LA volume. In this cohort, BMI was found to be directly and positively correlated with increased LA volume. CA has been shown to induce favorable remodeling in the LA in PeAF patients. For example, both PVI and fibrosis‐guided ablation have been found to be associated with LA volume, the most significant determinant of recurrence,^21^ decrease after CA.^22^ In our analysis, BMI was found to be strongly correlated with LA volume preablation, with increasing LA volume in each of our groups. However, BMI did not affect the extent of LA volume change expected with CA in our cohort. Also, BMI was found to not be associated with baseline fibrosis in our cohort, a finding highlighting the homogeneity of risk factor burden in our cohort. It also highlights the complexity and the heterogeneity of substrate development and manifestation in relation to the specific risk factors.

Given the higher rate of comorbidities, worse scar formation, and larger atrial volume, we expected to find a higher rate of ablation failure in obese patients. However, our analysis shows no significant different in ablation success among the different groups, showing that obese patients still retain benefit from CA even after persistence of AF. The literature on this is complicated. Many studies have shown that ablation in obese patients yields suboptimal results, while others showed no impact. For example, Cha et al. showed in a study on 532 patients with refractory AF who underwent CA had no difference in recurrence over a 1‐year follow‐up.^23^ A study by Jongnarangsin et al. illustrated that although obstructive sleep apnea was associated with recurrent AF after ablation of complex fractionated atrial electrograms regardless of atrial size, BMI was not associated with a shorter freedom from recurrence.^24^ On the other side, Tabaja et al. reported a retrospective analysis done on 5841 patients which showed that obesity group 3 is associated with increased AF recurrence, but BMI was not associated with increased complication rates or recurrence in the other groups.^25^ Interestingly, in this study, obstructive sleep apnea was not considered as a possible driver of increased recurrence in the morbidly obese patients.^25^

Many studies have shown that weight loss is associated with improved outcomes in AF patients such as ARREST‐AF, SORT‐AF, LEGACY, and the REVERSE‐AF studies.^26^, ^27^, ^28^, ^29^ Moreover, Donnellan et al. illustrated that bariatric surgery before ablation is associated with a higher ablation success rate in morbidly obese patients.^30^ One major confounder in these studies however is the intense risk factor, lifestyle modification, and follow‐up inherent to the intervention, which can lead to a cumulative improvement in risk factors regardless of BMI. Also, our study focuses on patients with more advanced and persistent disease. The effect of BMI on outcomes in PeAF patients has been lacking in the literature. PeAF patients usually have more advanced myopathy as marked by increased atrial fibrosis and enlarged atrial volume. Moreover, these patients have more complicated electrophysiological remodeling, explaining the lack of superiority with substrate modification in our patients. It could be that when atrial myopathy reaches this level, obesity already served its effect on atrial remodeling beforehand and is considered secondary. This notion of the point of no return has been suggested in the literature and has been shown even for weight loss interventions.^31^, ^32^, ^33^ For example, Mohanty et al. assessed the impact of weight loss in obese patients on ablation success in 90 patients prospectively. Unique to this study by Mohanty et al. compared to LEGACY and REVERSE‐AF, all patients were long‐standing PeAF patients. The results were interesting as they showed that although significant reduction in weight was attained, no impact on symptom severity and long‐term ablation outcome was observed.^32^

In summary, regarding the similar rate of AF recurrence across patient groups, multiple points can be made. First, this study is not the first to show that obesity does not affect outcomes in persistent AF patients. Patients with higher BMI in our study were ablated earlier than normal weight patients, which is possibly related to more intense follow‐up and can decrease ablation failure. Second, atrial myopathy is a multifaceted, complicated substrate, especially in persistent AF patients, and many factors should be considered in future studies, including atrial fibrosis arrhythmogenicity, which are still not possible to assess using current noninvasive techniques. Third, the increase in LA volume but not in atrial fibrosis hints toward the variety and complexity of the interaction between comorbidities and atrial myopathy, a space open to future research. Finally, the study was not powered to assess the differences among obese groups regarding ablation success. Nevertheless, CA in persistent AF population in still suboptimal and extra‐pulmonary substrate is still difficult to target successfully. Our study, among others, shows possible mechanisms that should be investigated more in the future for optimizing substrate modification in the future and individualizing ablation based on comorbidity profile. These mechanistic insights can lay the ground for future research that will directly impact clinical practice on a day‐to‐day basis and improve outcomes of ablation in patients with persistent AF.

## 5 CONCLUSION

In PeAF patients, patients with more ablation‐induced scar formation had less recurrence. Increased BMI is significantly correlated with less scar formation, less coverage of fibrosis, and higher residual fibrosis. Nevertheless, obese PeAF did not have a lower success rate in our cohort.

## 6 STUDY LIMITATIONS

The current subanalysis has multiple limitations. First, this is retrospective analysis of the DECAAF II trial, which inherently holds with it the limitations associated with the retrospective design. Second, the study was performed across 44 centers globally, meaning that operators had varying skills and expertise which could have affected the outcomes and scar formation, especially in the fibrosis‐guided arm. Moreover, although CMR protocols were standardized, the CMR machines varied between study sites, which could have led to some variability although the CMR protocols were standardized. Third, the follow‐up period was relatively short (12–18 months) which might have influenced the assessment of ablation success in this cohort. Additionally, the use of BMI and obesity classifications comes with some general limitations not specific to our work. BMI has faced scrutiny as a metric to evaluate obesity and techniques such as a visceral adipose measurement may be more efficacious.^34^ The CDC cutoff values defining each BMI group are somewhat arbitrary and studies have shown advantages to different delineations. Finally, our study assumed negligible changes in BMI throughout the study.

## Supporting information

Supporting information.

## Data Availability

The data underlying this article will be shared upon reasonable request to the corresponding author.
