# Supplementary material for: Body mass index as a determinant of scar formation post‐AF ablation: Insights from DECAAF II
Source: J Cardiovasc Electrophysiol. 2024 Sep 30;35(12):2330–41. doi: 10.1111/jce.16448 (PMC11650536; doi:10.1111/jce.16448)
Supplement: Supplementary file 1 — Supporting information. [file JCE-35-2330-s001.docx]

**Supplementary Material :**

**Supplementary table S1: Multivariable Analysis for Conventional PVI Arm Only**

| **Multivariate Linear Regression for population within Conventional PVI** | | | |
| --- | --- | --- | --- |
| **Follow-Up Ablation Scar with Multiple Covariates** | | | |
|  | Coefficient | Standard Error | P-value |
| (Intercept) | 11.228 | 3.217 | 0.001 |
| BMI^a^ | -0.124 | 0.051 | 0.017 |
| Age | 0.012 | 0.035 | 0.740 |
| (Sex)Male | -0.344 | 0.838 | 0.681 |
| LA^c^ volume | -0.006 | 0.009 | 0.524 |
| Baseline Fibrosis | -0.019 | 0.045 | 0.673 |
| CRP^b^ | 0.047 | 0.069 | 0.497 |
| Diabetes Mellitus | -0.721 | 1.158 | 0.535 |
| Congestive Heart Failure | 2.699 | 0.873 | 0.002 |
| Hypertension | 0.678 | 0.678 | 0.319 |
| Coronary Artery Disease | 3.150 | 1.341 | 0.020 |
| Peripheral Vascular Disease | -2.683 | 1.496 | 0.075 |
| History of Stroke | 1.915 | 1.246 | 0.126 |
| Aldosterone Inhibitors | -1.149 | 1.059 | 0.280 |
| Antiarrhythmic Medications | 0.799 | 0.630 | 0.206 |
| Impedance Drop | 0.075 | 0.116 | 0.516 |

*Abbreviations: ^a^BMI, body mass index; ^b^CRP, C-Reactive protein; ^c^LA, left atrium*

**Supplementary table S2: Multivariable Analysis for PVI + Fibrosis-guided Arm only**

| **Multivariate Linear Regression for population within PVI + Fibrosis-guided ablation** | | | |
| --- | --- | --- | --- |
| **Follow-Up Ablation Scar with Multiple Covariates** | | | |
|  | Coefficient | Standard Error | P-value |
| (Intercept) | 5.782 | 4.270 | 0.178 |
| BMI^a^ | -0.148 | 0.072 | 0.040 |
| Age | 0.099 | 0.051 | 0.053 |
| Male Gender | 1.797 | 1.046 | 0.088 |
| LA volume^b^ | -0.016 | 0.010 | 0.127 |
| Baseline Fibrosis | 0.105 | 0.060 | 0.081 |
| C-reactive Protein | -0.135 | 0.053 | 0.013 |
| Diabetes Mellitus | 0.712 | 1.473 | 0.629 |
| Congestive Heart Failure | 1.193 | 0.970 | 0.220 |
| Hypertension | -0.045 | 0.890 | 0.960 |
| Coronary Artery Disease | -1.750 | 1.455 | 0.231 |
| Peripheral Artery Disease | 0.023 | 1.617 | 0.989 |
| History of Stroke | 0.094 | 1.613 | 0.954 |
| Aldosterone Inhibitor use | -0.933 | 1.473 | 0.527 |
| History of antiarrhythmic Medication | -0.048 | 0.780 | 0.951 |
| Impedance Drop | 0.404 | 0.152 | 0.009 |
| **Residual Fibrosis with Multiple Covariates** | | | |
|  | Coefficient | Standard Error | P-value |
| (Intercept) | -0.847 | 1.572 | 0.591 |
| BMI^a^ | 0.056 | 0.026 | 0.034 |
| Age | -0.021 | 0.019 | 0.264 |
| Male Gender | -0.584 | 0.385 | 0.131 |
| LA^b^ volume | 3.42E-04 | 0.004 | 0.930 |
| Baseline Fibrosis | 0.801 | 0.022 | <0.001 |
| C-Reactive Protein | 0.049 | 0.020 | 0.014 |
| Diabetes Mellitus | -0.124 | 0.542 | 0.819 |
| Congestive Heart Failure | 0.163 | 0.357 | 0.649 |
| Hypertension | -0.024 | 0.328 | 0.942 |
| Coronary Artery Disease | 0.441 | 0.536 | 0.411 |
| Peripheral Vascular Disease | 0.271 | 0.595 | 0.650 |
| History of Stroke | 0.263 | 0.594 | 0.658 |
| Aldosterone Inhibitor Use | 0.248 | 0.542 | 0.648 |
| Antiarrhytmic Medications | 0.084 | 0.287 | 0.771 |
| Impedance Drop | -0.038 | 0.056 | 0.502 |
| **Covered Fibrosis with Multiple Covariates** | | | |
|  | Coefficient | Standard Error | P-value |
| (Intercept) | 0.288 | 0.092 | 0.002 |
| BMI^a^ | -0.004 | 0.002 | 0.022 |
| Age | 0.001 | 0.001 | 0.303 |
| Male Gender | 0.021 | 0.023 | 0.354 |
| LA^b^ volume | 2.09E-04 | 0.000 | 0.360 |
| Baseline Fibrosis | -0.003 | 0.001 | 0.012 |
| C-reactive Protein | -0.002 | 0.001 | 0.119 |
| Diabetes Mellitus | 0.011 | 0.032 | 0.736 |
| Congestive Heart Failure | -0.007 | 0.021 | 0.739 |
| Hypertension | 0.001 | 0.019 | 0.951 |
| Coronary Artery Disease | -0.035 | 0.031 | 0.267 |
| Peripheral Vascular Disease | -0.019 | 0.035 | 0.598 |
| History of Stroke | -0.009 | 0.035 | 0.796 |
| Aldosterone Inhibitor Use | -0.017 | 0.032 | 0.596 |
| Antiarrhythmic Medication | -0.016 | 0.017 | 0.332 |
| Impedance Drop | 0.006 | 0.003 | 0.096 |

*Abbreviations: ^a^:BMI, body mass index; ^b^:LA, left atrial*
